# HPV Vaccination: An Underused Strategy for the Prevention of Cancer

**DOI:** 10.3390/curroncol29050303

**Published:** 2022-05-23

**Authors:** Gilla K. Shapiro

**Affiliations:** 1 Department of Supportive Care, Princess Margaret Cancer Centre, 610 University Ave, Toronto, ON M5G 2C1, Canada; gilla.shapiro@uhnresearch.ca; 2 Global Institute of Psychosocial, Palliative and End-of-Life Care (GIPPEC), University of Toronto and Princess Margaret Cancer Centre, 700 Bay Street, Suite 2303, Toronto, ON M5G 1Z6, Canada

**Keywords:** human papillomavirus, cancer prevention, vaccine uptake, vaccine hesitancy, behavioural and social drivers of vaccination framework

## Abstract

Human papillomavirus (HPV) vaccination prevents cervical, head and neck, and anogenital cancers. However, global HPV vaccine coverage falls short of global targets and has seen unexpected and dramatic declines in some countries. This paper synthesizes the impact of HPV on the global burden of cancer and the potential benefit of HPV vaccination. Approximately 5% of the world’s cancers are specifically attributed to HPV. While the greatest global burden of HPV is cervical cancers in low- and middle-income countries, HPV-associated head and neck cancers are increasing in high-income countries and have surpassed cervical cancer as the primary HPV-associated cancer in some countries. Therefore, it is also critical to improve gender-neutral HPV vaccination. Understanding the modifiable drivers of vaccine acceptance and uptake is important for increasing HPV vaccination. The Behavioural and Social Drivers of Vaccination framework is broadly applied to identify key factors associated with HPV vaccination including domains concerning practical issues, motivation, social processes, and thinking and feeling. Among the behavioural strategies available to reduce the incidence and mortality of cancer, increasing HPV vaccination stands out as having unrealized potential to prevent disease, financial cost, and psychological distress. An understanding of the shifting burden of HPV and the factors associated with vaccination can be leveraged to regularly measure these factors, develop interventions to promote vaccine uptake, and improve global HPV vaccine coverage. Future research in diverse contexts is necessary to investigate the barriers and facilitators of global HPV vaccination.

## 1. The Burden of HPV

The impact of specific health behaviours in causing cancer, such as drinking alcohol, smoking cigarettes, and prolonged sun and other radiation exposure, are well recognized. It is not as well known in the general public that an important proportion of the global cancer burden is associated with infectious agents including viruses, bacteria, and parasites [1,2]. Millions of cancers worldwide are caused annually by infectious agents such as human papillomavirus (HPV), hepatitis B virus, hepatitis C virus, *Helicobacter pylori*, and the Epstein–Barr virus [1,3]. Chief among infections that cause cancer is HPV.

HPV is the most common sexually transmitted infection worldwide [4,5,6,7,8]. The majority of sexually active people will contract HPV during their lifetime (approximately 75–80%) [9]. While most HPV infections (70–90%) are asymptomatic and will resolve on their own within 1–2 years, persistent infection (or multiple reinfections) can cause morbidity and mortality [10,11]. There are around 200 different genotypes of HPV, of which more than 20 are known or probable carcinogens [11,12].

Oncogenic strains of HPV (predominantly HPV 16/18, so-called “high-risk” strains) cause almost all cervical cancers [13]. However, the burden of HPV extends beyond cervical cancers. HPV also causes oropharyngeal (mouth, throat, tongue, and tonsils), vaginal, vulvar, penile, and anal cancers [13]. Research suggests that HPV is also associated with sinonasal, conjunctiva, and lacrimal sac cancer [14,15,16]. A rare but serious consequence of HPV infection is recurrent respiratory papillomatosis, whereby HPV infection can be transmitted by maternal HPV infection as well as individual sexual behaviours [17]. HPV infection has also been associated with a higher risk of HIV acquisition [18]. In addition, two strains of HPV (HPV 6/11, i.e., so-called “low risk” strains) are responsible for 96–100% of anogenital warts [19]. While not deadly, anogenital warts can impact one’s quality of life and accrue substantial financial costs to health care systems [20].

It is estimated that 4.5–5.2% of global cancers are attributed specifically to HPV [6,10,11,21], which represents upwards of 630,000 new cancer cases annually [1,21]. However, the prevalence and persistence of HPV infection varies by geographical region, sex, age, ethnicity, anatomical location of the infection, having a weakened immune system, and health behaviours (such as number of sexual partners, sexual practices, or tobacco use) [4,6,22,23]. For example, HPV infections represent 8.6% of all cancers in females and 0.8% of all cancers in males [21]. Prevalence of HPV infections is elevated in specific minority groups, including individuals with HIV infection and men who have sex with men (MSM) [24].

The greatest global burden of HPV-associated cancers is cervical cancer in low- and middle-income countries (LMIC); HPV-associated cancers represent 6.7% of all cancers in LMIC and 2.8% in high-income countries [1,21,25]. However, in high-income countries (HIC), HPV infection is a major and increasing cause of head and neck cancers in both males and females [1,11,21,26,27], although it is increasing at a faster rate among males [8,12,28]. In HIC, where there are vaccination and screening programs for cervical cancer, oropharyngeal cancers are the most common HPV-associated cancer and represent a higher burden than in LMIC [12,26,29,30]. The increasing incidence of HPV-associated oropharyngeal cancers in HIC is partly due to a higher frequency of oral sex, a greater number of sexual partners, a reduction in tobacco use, and the improvement in HPV detection [27,31].

The psychosocial impacts of HPV-associated cancers are an underappreciated burden. Similar to other patients with cancer, individuals who develop HPV-associated cancers can experience physical and psychological concerns associated with their diagnosis and treatment. However, in addition, individuals may also experience distress related to the cause and transmission of HPV [32]. The acknowledgment that one’s cancer is derived from a sexually transmitted infection can be upsetting and confusing. Individuals may wonder how they contracted HPV, experience body image issues, have concerns about increased cancer risk for their sexual partners, or not engage in sexual intimacy for fear of transmitting HPV [33]. For instance, one exploratory study found that 14% of patients initiating radiotherapy for a newly diagnosed oropharyngeal cancer reported that they kept their HPV status secret for fear of embarrassment, stigma, or privacy [8,33]. In addition, given the benefit of some preventative measures, individuals may experience self-blame, guilt, or shame if they did not engage in these health behaviours, or confusion or anger if they took preventative measures and nevertheless developed cancer. Greater research is required to better understand the psychological distress in patients who have HPV-associated cancers and what therapeutic approaches best alleviate this distress [8]. It is important to conduct distress screening and provide psychotherapy and psychoeducation to support patients with emotional coping, increase understanding of their condition, and promote effective couple communication and support (as appropriate) [8,34].

## 2. Vaccination as a Tool to Prevent Cancer

Cancers caused by HPV are largely preventable [35]. Given the global burden of HPV, primary and secondary prevention has been emphasized. While pap screening and HPV DNA testing are available to detect cervical cancers in early stages (i.e., secondary cancer prevention methods), there is no comparable screening measure for HPV infections for other cancers, including head and neck cancers. Initial efforts for screening for other malignancies require more research to improve early detection of HPV [29], but this is particularly challenging given the rarity of these other cancers and to-date unknown precursor lesions [36]. However, primary prevention through HPV vaccination is available and is estimated to have the potential to prevent 70% to 90% of all HPV-related cancers [11].

Since 2007, four prophylactic (i.e., preventative) vaccines have been developed to protect against future HPV infections, specifically including Gardasil^®^, Cervarix^®^, Gardasil^®^9, and Cecolin^®^ vaccines (Table 1) [7,12,37,38,39,40,41]. Given that vaccines can prevent but not treat HPV infections, vaccines are recommended primarily for younger individuals before potential exposure to HPV. The vaccines differ in the number of strains of HPV that they target (Table 1). Therefore, not all HPV-related malignancies can be prevented through these available vaccines. As vaccine technology advances, new HPV vaccines are likely to be introduced and incorporated into immunization programs that will be able to provide additional protection. Notably, therapeutic HPV vaccines are being researched for head and neck cancer, with clinical trials underway that have found a positive immune response to the vaccines [42].

Until April 2022, the World Health Organization (WHO) recommended two doses of the HPV vaccine for girls aged 9–14. Given increasing evidence that single-dose schedules provide comparable efficacy to two or three doses, the WHO Strategic Advisory Group of Experts on Immunization (SAGE) recently recommended a one- or two-dose schedule for girls and young women who are 9–20 years old [43]. Girls and young women who have a compromised immune system, including those living with HIV, continue to be recommended to receive three doses of the HPV vaccine if feasible, and if not, at least two doses [43]. HPV vaccination has recognizable benefits for males and females (see above), and some countries with the resources have chosen to offer gender-neutral vaccination programs. However, debate persists regarding whether HPV vaccination programs should also target boys [31]. Some experts question whether the prevalence of HPV infection in boys merits intervention and argue that through herd protection effects, HPV vaccination programs that target girls will also benefit many boys and be a more cost-effective option [44]. However, a female-only approach to vaccination relies on herd protection and does not consider low or a sudden drop in female HPV vaccine uptake rates (preventing herd effects). Furthermore, female-only vaccination does not protect MSM, who experience a high burden of anal cancer and anogenital warts [45], or heterosexual men with sexual partners who chose to not be vaccinated or came from a country that did not have an HPV immunization program [46].

Research has demonstrated that HPV vaccines are safe and effective in reducing HPV-related infections, genital warts, and pre-cancers [39,47,48,49,50,51,52]. Clinically effective protection and sustained antibody titers have been demonstrated for at least 10 years after vaccination [53]. In terms of the HPV vaccine’s real-world impact, epidemiological evidence has found significant reductions in HPV-related infections [39,54,55,56]. For example, a systematic review and meta-analysis of the population-level impact of HPV vaccination programs found that , there was a significant decrease in HPV 16/18 infections (by 68%) and anogenital warts (by 61%) in countries with female vaccine coverage of at least 50% [50]. A recent study that examined 1.67 million Swedish girls and women from 2006 to 2017 found a substantially reduced risk of invasive cervical cancer at the population level [57].

Areas that would benefit from additional research on HPV vaccine effectiveness include further investigations on the nonavalent vaccine, the quadrivalent vaccine in males, reduced dose schedules, and in specific population groups [45]. Additionally, more time is needed to investigate the full benefit of HPV vaccines for head and neck cancers [36], particularly given the long interval between HPV infection and the development of oropharyngeal cancers [58]. Nevertheless, burgeoning evidence has found a significant decrease in vaccine-type oral or oropharyngeal HPV infections in study participants who were immunized with HPV vaccines, which is suggestive of the potential of HPV vaccines for the prevention of these cancers [26,59].

In terms of vaccine safety, serious adverse events due to the HPV vaccines are rare, and most injection-site reactions are pain and swelling at the vaccine injection site [48,60,61]. The safety of the HPV vaccines is endorsed by the World Health Organization, the Centers for Disease Control and Prevention, the National Advisory Committee on Immunization, and other international immunization advisory committees [62,63,64].

HPV vaccination is also a cost-effective public health strategy; especially among girls and in settings where cervical cancer screening is low [65,66]. Models of HPV vaccination that included both boys and girls have also demonstrated cost-effectiveness when they took into consideration all HPV-related diseases, such as the burden of HPV-associated head and neck cancers, and the suboptimal coverage of vaccination in females [46,67]. A recent systematic review of the cost-effectiveness of HPV vaccines, which also included non-cervical HPV-associated disease, found that female-only vaccination strategies were 2.85 times more cost-effective, while gender-neutral vaccination was 3.89 times cost effective [67].

## 3. The Underuse of HPV Vaccination

Despite the benefit of HPV vaccination, global vaccine coverage is not reaching the targets required to provide herd immunity. Models predict that elimination of HPV infections requires global vaccination rates of 80% [31] and the WHO Cervical Cancer Elimination strategy aims to increase HPV vaccination to 90% of all adolescent girls by 2030 [68]. However, in 2020, the average completion rates across WHO regions ranged from 29% to 60% [69]. Rates vary by country. For example, in some HIC, such as Australia and the United Kingdom, school-based programs have reached 70% to 80% of girls for the final dose [70]; however, in other HIC, such as France, coverage has not reached 50% [71,72]. HPV vaccine coverage is also lower compared to other routine vaccines in younger children [73]. In some countries where gender-neutral vaccination is available, fewer boys than girls have received the HPV vaccine, and this has potential consequences for the prevention of HPV-associated head and neck cancers [73,74,75].

While some countries, such as the United States, have reported increased HPV vaccine coverage over time [76], other countries have experienced instability or unexpected declines in HPV vaccine coverage (unrelated to the COVID-19 pandemic). For example, in 2013, Japan’s government removed a proactive recommendation for HPV vaccines due to unconfirmed reports of safety concerns that appeared in Japanese media [77]. As a result, HPV vaccine coverage in Sapporo (a city in North Japan with a population of almost 2 million people) plummeted from approximately 70% to less than 1% [77]. Although some countries have managed to obtain consistently high HPV vaccine uptake rates [78], Japan’s situation is not an isolated occurrence. Other countries, including Colombia, Denmark, and Ireland, have experienced sudden drops in HPV vaccine coverage despite initially high uptake rates [79,80,81,82].

## 4. Drivers for Achieving High Global HPV Vaccine Coverage

To better understand the drivers for achieving vaccine coverage, the WHO established the “Measuring Behavioural and Social Drivers of Vaccination” (BeSD) working group, in 2018 [83,84,85,86]. The group, comprised of global health experts, developed the BeSD framework to systematically gather and utilize data on the behavioural and social drivers of vaccine uptake [83,84,85]. The framework includes four domains that are modifiable contributors to vaccine uptake. [83,84,85,86]. The BeSD framework is broadly applied here to identify key drivers for achieving high global HPV vaccine coverage, and includes domains concerning practical issues, motivation, social processes, and thinking and feeling.

### 4.1. Practical Issues

There are practical issues that influence HPV vaccination such as the availability and accessibility of the vaccine. One challenge has been its availability in national immunization programs. As of March 2022, only 117 countries of 194 WHO Member States (i.e., 60%) had introduced HPV vaccination for girls in their national immunization schedules, and since 2013, only 38 countries had included boys (20%) [69]. LMIC introduced HPV vaccination in national programs at much slower rates than HIC, reflecting and perpetuating global disparities in HPV-related cancer incidence and mortality [73]. There are also limitations in the affordability of the HPV vaccines, particularly in LMIC which do not receive support from Gavi, the Vaccine Alliance, a global health partnership that helps deliver vaccines [87,88]. This has raised debates, similar to the COVID-19 vaccines, about the tension between national priorities in HIC to provide broad vaccination programs to protect their population (e.g., providing HPV vaccines to boys or providing COVID-19 vaccines to young children) and achieving global immunization goals of protecting the most at-risk populations.

Worldwide shortages of HPV vaccines have impacted some programs’ ability to deliver vaccinations [73,87]. In addition, similar to other routine immunizations [89], HPV vaccination was impacted by program disruptions due to the COVID-19 pandemic [73]. For example, in England, HPV vaccination in 2020–2021 had increased from 2019–2020, but were not yet consistently reaching pre-pandemic rates, and those who have missed HPV vaccinations had not caught up by 2021 [90]. The longer-term effects of this under-vaccinated cohort is far reaching, and rapid recovery is crucial to prevent future excess cancer burden [73].

School-aged children and adolescents are often more difficult for immunization programs to reach (compared to the vaccination of younger children) and require specialized strategies that enhance access to all groups. Parents/caregivers (hereafter “parents”) and their school-aged children must know where and how to access the HPV vaccine. To improve ease of access, school-based HPV vaccine programs have demonstrated benefits in increasing vaccine uptake and equity [91,92]. Although some LMIC countries, such as Rwanda, have achieved high HPV vaccine coverage using a school-based strategy, many LMIC do not have funded school health programs, and providing HPV vaccination in schools can be expensive and unfeasible [73]. For example, school programs require additional coordination, resources for health workers’ transport and daily per diems [93]. Challenges in school-based programs include loss to follow-up due to family migration or school transfers, and creative strategies are needed to catch-up children who have missed doses. In addition, combining HPV vaccination with other health services, as South Africa and Rwanda have done, can save costs and improve access [93]. A simpler vaccine schedule with fewer doses, as recently recommended by SAGE [43], may also improve ease of access and HPV vaccination, particularly in LMIC.

### 4.2. Motivation

**Figure 1:**
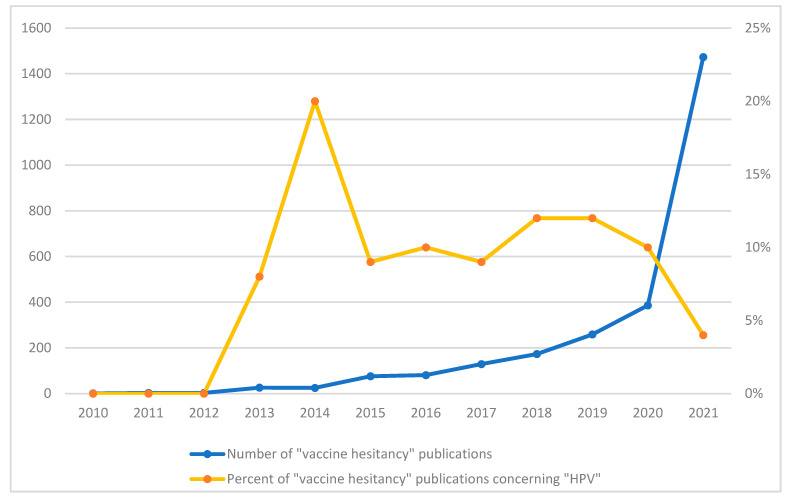
Number of vaccine hesitancy publications by year, and percent concerning HPV. *Note*. This figure was modified from previous work by the author [98]. Pubmed was searched for articles published between 2010 and 2021 using the term “vaccine hesitancy”, and the number of papers is plotted against the year (in blue, see left y-axis). A separate search in Pubmed over the same time was conducted using the terms “vaccine hesitancy” AND “HPV”; the percent of “vaccine hesitancy” papers concerning “HPV” was calculated (in orange, see right y-axis).

Motivation includes individuals’ intention, willingness, and hesitancy to get vaccinated. Although individuals have been conflicted or opposed to receiving vaccinations since modern inoculation was introduced 200 years ago [94,95,96,97], the use of the term “vaccine hesitancy” to describe this phenomenon is a relatively new and suddenly ubiquitous construct. The increased popularity of this term is evident by the burgeoning number of publications using this construct, as evidenced by a search conducted by this author (see Figure 1). While the literature base on “vaccine hesitancy” increased from 2010–2021, it exploded in association with the COVID-19 pandemic. Not surprisingly, most (77%) of the published literature on vaccine hesitancy focused on COVID-19 in 2021. The percent of publications on vaccine hesitancy that focused specifically on HPV peaked in 2014 (at 20%), but has averaged around 9% over the past decade.

HPV vaccine hesitancy is defined here to refer to being conflicted about, or opposed to, getting the HPV vaccine; it reflects individual willingness or intention towards vaccination [83]. The definition of vaccine hesitancy as a motivational state contrasts with the previously held predominant understanding of vaccine hesitancy as an attitude [83]. This definition also separates vaccine hesitancy from vaccine behaviour (i.e., vaccine uptake or completion) and allows these constructs to be measured separately [83]. Intention to receive the HPV vaccine has been found to be a predictor of vaccine uptake [99], although more detailed research is needed to better understand this relationship in diverse contexts [100].

### 4.3. Social Processes

Social norms and influence—including by one’s family, friends, healthcare provider, and religious or community leaders—impact motivation to receive the HPV vaccine. Notably, a healthcare provider’s recommendation has consistently been recognized as a critical factor for HPV vaccination [101,102], and physician-focused interventions (such as education and training, audit and feedback and/or electronic decision support or alerts) increase HPV vaccine uptake [103]. For example, a systematic review of 59 eligible studies from the United States (of 265,083 patients) found that receiving a healthcare provider’s recommendation was associated with HPV vaccine initiation (with a pooled odds ratio of over 10) [104]. Other relationships in one’s social circle can influence parents’ attitudes both for and against vaccination [79,105]. Religious and community leaders (including leaders of school boards) also seem to play an important role in vaccine motivation and acceptance [46,91,106,107], but these relationships require greater investigation in different contexts.

In many countries, HPV vaccination is voluntary, and consent is required from the child and/or the child’s parent, depending on the child’s age and the country’s guidelines for consenting to vaccines [92]. In contrast to vaccinating infants or young children, school-aged children therefore have the ability to participate in their own vaccine decision. Parents’ favourable views and discussion with their child have been associated with HPV vaccination [99,100]. However, qualitative reports of 262 adolescent-parent dyads suggest a lack of alignment between parents and adolescents regarding who made the decision to receive the HPV vaccination [108]. In some contexts, parents report that they would like to be greatly involved in the decision for their child to receive the HPV vaccine [109]. It is pertinent that immunization programs consider the need for information, preferences for communication, and joint decision making among children, parents, and communities [93].

In considering the broader social context, some research has examined the impact of online social networks and media on HPV vaccine intentions and uptake. One study found that as little as 5 to 10 min of access to vaccine-critical websites influenced participants’ risk perception and vaccine intentions [110]. Similarly, compared to a control group, another study reported that participants who were exposed to online content depicting a negative outlook on HPV vaccines then perceived the vaccine as less safe, held more negative attitudes, and reported reduced vaccine acceptance [111]. Some studies have used health informatics to examine the impact of reading negative information about vaccines online using big data. For example, a study of over 250,000 tweets related to the HPV vaccines in the United States (between 2013 and 2015) found that vaccine coverage was lower in states where there was a higher proportion of exposure to safety concerns, misinformation, and conspiracies, which suggests that negative representations of vaccines in the media is associated with (either reflecting or influencing) HPV vaccination [112]. A recent population-based retrospective cohort study of all girls born in Denmark from 1997 to 2006 had similar findings; where periods of extensive negative media coverage were associated with substantially reduced HPV vaccination [82]. The introduction of a national information campaign (alongside a catch-up program) was associated with some recovery in vaccination rates, but still left many older girls unvaccinated who may have otherwise received the vaccine [82]. These studies highlight that a quick and proactive response to managing public concerns is critical and may include tracking public sentiment and social media, having a social media presence, providing accurate information, and using evidence-based communication methods.

### 4.4. Thinking and Feeling

Psychological factors—including knowledge, values, attitudes, and beliefs—also influence individuals’ motivation to receive the HPV vaccine. Changing policies regarding HPV vaccines (e.g., the administered vaccine, number of recommended doses, and groups targeted for vaccination) can make it a challenge to establish consistent population-level knowledge about the HPV vaccines. For example, it is possible that an initially female-only strategy created confusion and compliance issues for male vaccine uptake (where available). Overall, knowledge of HPV-related diseases and the HPV vaccine is often found to be variable or low [113,114]. However, there is mixed evidence on whether low knowledge is associated with decreased vaccine acceptance and uptake [100]. This could be because knowledge is multifaceted, and certain aspects of vaccine knowledge (e.g., that the HPV vaccine is recommended for males) may be more important for initiating vaccination, while other knowledge gaps (e.g., the HPV vaccine being part of a multi-dose series) may be less important given available systematic supports (e.g., reminders). It is also possible that the evidence is mixed because many studies compare the knowledge of HPV vaccinated and non-vaccinated groups and do not consider more nuanced stages of vaccine decision making [115]. Research that has used the Precaution Adoption Process Model [116] to identify multiple stages of HPV vaccine decision making has shown a more complex relationship between knowledge and vaccination [102,115]. For example, one study by this author and colleagues found *lower* HPV vaccine knowledge in parents in earlier stages of decision making (i.e., parents who were unaware of the HPV vaccine or unengaged about whether to vaccinate their child, as compared to parents whose child was vaccinated), but *higher* HPV vaccine knowledge in parents who decided not to vaccinate their child (as compared to vaccinated) in multivariate analyses [102].

Regarding specific vaccine attitudes and beliefs of import, the perceived benefit of HPV vaccination for preventing cancer and other diseases has been consistently related to HPV vaccine acceptability and uptake [99,102,117,118]. Furthermore, notable attitudes and beliefs that contribute to HPV vaccine hesitancy include lack of confidence, lack of trust in vaccination programs and providers, concerns about vaccine safety, and concerns about vaccine side effects [99,102,117,118,119,120,121]. While attitudes and beliefs about the HPV vaccines share these similarities to other vaccines in younger children, there are also unique aspects of the HPV vaccine that impact attitudes and beliefs, including it being a vaccine for school-aged children, a relatively newer vaccine, a vaccine for a sexually transmitted infection, and a vaccine that is used to prevent cancer. For example, research has found a delay in HPV vaccination related to parents and providers waiting until a child is “about to be sexually active” [31]. Despite a lack of evidence [122], there have been concerns raised by religious leaders and parents that vaccinating children against HPV could provide children with permission to engage in risky sexual behaviors [123]. Individuals can also hold multiple and incongruent beliefs at one time such as thinking a child is at risk for an HPV-associated cancer and thinking that the HPV vaccine is not safe. The interplay of psychological factors, particularly in the context of other variables, requires further research.

## 5. Conclusions

HPV vaccination stands out as having unrealized potential to prevent cancers, financial cost, and psychological distress. HPV causes cervical cancer and is a substantial burden for LMIC, while HPV-associated head and neck cancers are also a leading and increasing priority for HIC. Several HPV vaccines have been developed with substantial research demonstrating their effectiveness, safety, and cost-effectiveness. While both LMIC and HIC have shown that it is feasible to achieve high HPV vaccination, global HPV vaccine coverage is not consistently reaching targets and has been further impacted by program disruptions caused by the COVID-19 pandemic [73].

This synthesis, while not exhaustive, broadly applies the BeSD framework to understand modifiable drivers of HPV vaccine acceptance and uptake. An understanding of the shifting burden of HPV and the factors associated with HPV vaccination can be leveraged to regularly measure these factors, develop interventions to promote vaccine acceptance, and improve global HPV vaccine coverage. Future research in diverse populations is needed to comprehensively evaluate these aspects. This requires comparative measurement of these factors across countries and over time.

## Figures and Tables

**Table 1:** Available vaccines to prevent HPV-associated cancers.

**Brand Name**	Gardasil^®^	Cervarix^®^	Gardasil^®^9	Cecolin^®^
**Developer**	Merck	Glaxo SmithKline	Merck	Xiamen Innovax Biotech
**Date licensed**	2006	2007	2014	2020 *
**Type of vaccine**	Quadrivalent	Bivalent	Nonavalent	Bivalent
**HPV strains targeted**	6/11/16/18	16/18	6/11/16/18/31/33/45/52/58	16/18

* Licensed in China in 2020 and currently under review by the World Health Organization [41].

## Data Availability

Data presented in this paper are available on request from the author.
